# R program for estimation of group efficiency and finding its gradient. Stochastic data envelopment analysis with a perfect object approach

**DOI:** 10.1016/j.dib.2018.06.097

**Published:** 2018-07-03

**Authors:** Alexander Vaninsky

**Affiliations:** City University of New York, Hostos Community College, USA

## Abstract

The data presented here are related to the research article “Energy-environmental efficiency and optimal restructuring of the global economy” (Vaninsky, 2018) [1]. This article describes how the world economy can be restructured to become more energy-environmental efficient, while still increasing its growth potential. It demonstrates how available energy-environmental and economic information may support policy-making decisions on the atmosphere preservation and climate change prevention. This Data article presents a computer program in R language together with examples of input and output files that serve as a means of implementation of the novel approach suggested in publication [1]. The computer program utilizes stochastic data envelopment analysis with a perfect object (SDAEA PO) to calculate the group efficiency of a collection of decision-making units (DMUs), the efficiency gradient, and the projected gradient. The projected gradient is computed in the case when the SDEA PO inputs and outputs are given as shares in total, to satisfy the constraints of adding up to a unit. By so doing, the program assesses the energy-environmental efficiency of the global economy and determines the ways of its maximum possible increase via locally optimal economic restructuring.

**Specifications Table**

**Table:** 

Subject area	*Environmental economics, Energy economics, Economics of global economy.*
More specific subject area	*Economic restructuring.*
Type of data	*R program script, input file, output file.*
How data was acquired	*The R program was developed by the author. Input file contains data calculated by the author based on the information collected from the website of the U.S. Energy Information Administration*www.eia.gov*. Output file results from the computations.*
Data format	*The R program script is a text file; input and output files are csv files.*
Experimental factors	*N/A*
Experimental features	*Using the data contained in the input file, the program calculates group efficiency score, its gradient, and projected gradient. The obtained results demonstrate possible applications of the suggested approach.*
Data source location	*U.S. Energy Information Administration website:*www.eia.gov.
Data accessibility	*The input data were calculated based on the information publicly available at*www.eia.gov*as shown in Tables 1 and 2 in*[1].
Related research article	*A. Vaninsky, Energy-environmental efficiency and optimal restructuring of the global economy, Energy 153 (2018) 338–348.*

**Value of the data**

• A combination of a theoretical framework and a computer program forms a tool for the analysis and development of environmentally friendly policies.
• The computer program estimates the energy-environmental efficiency of the global economy and determines the ways of its optimal increase.
• The computer program allows for analysis of the group efficiency of different economic systems.

## 1 Data

This article presents an R program that utilizes and further develops a novel stochastic data envelopment analysis with a perfect object method (SDEA PO) introduced in Vaninsky [2]. The program implements an algorithm invented in Vaninsky [1] aimed at energy-environmental efficient restructuring of the global economy. Examples of the input and output files are provided. The input file contains shares of the main national and regional economies in gross domestic product (GDP), clean energy consumption, carbon dioxide emissions (CO2), and population, respectively. The output file comprises the group efficiency score, its gradient, and projected gradient. The output data determine the direction of optimal energy-environmental economic restructuring.

## 2 Experimental design, materials, and methods

This program advances a novel SDEA PO method introduced in [2] by using the approach proposed in the related article. The SDEA PO considers Decision Making Units (DMUs) as occurrences of a universal DMU characterized by distributed inputs and outputs. The computer program calculates the group efficiency score, its gradient, and the projected gradient. A part of the program that calculates the group efficiency index is borrowed from [2]. Manual input contains the input file name and path, output characterization, and the number of outputs, given as the values of the variables inp_filename, inp_path, info, and n_DEA_outputs, correspondingly. Total number of inputs and outputs is determined automatically by the program, and a number of SDEA PO inputs is calculated as the difference between the total and the number of outputs. This version of the program assumes two outputs and two inputs. In case of different numbers, the formulas for the probability distributions should be adjusted in the lines labeled “# --- 2 inputs” or “# --- 2 outputs”, respectively, in the same structure as shown in [2]. An input csv file contains shares of the gross domestic product (GDP), clean energy consumption, carbon dioxide emissions (CO2), and population for the main national and regional economies as shown in Table 4 of the related article. An output csv file comprises the group energy-environmental efficiency score and the components of its gradient and projected gradient. The SDEA PO outputs should precede inputs. The input data correspond to the entries in columns (2) and (3) of Table 1, followed by the entries in columns (2) and (3) of Table 2, and then by the entries in column (6) of Table 1 in the related article, correspondingly. The precision of the input data should be at least 9 decimal places. An output file has a literal “-output” added to the input file name.

## References

[1] Vaninsky A. (2018). Energy-environmental efficiency and optimal restructuring of the global economy. Energy.

[2] Vaninsky A. (2013). Stochastic DEA with a perfect object and its application to analysis of environmental efficiency. Am. J Appl. Math. Stat..

