# Supplementary material for: R program for estimation of group efficiency and finding its gradient. Stochastic data envelopment analysis with a perfect object approach
Source: Data Brief. 2018 Jul 3;19:1844–6. doi: 10.1016/j.dib.2018.06.097 (PMC6141417; doi:10.1016/j.dib.2018.06.097)
Supplement: Supplementary file 1 — Supplementary material [file mmc1.doc]

We wish to confirm that there are no known conflicts of interest associated with this publication and there has been no significant financial support for this work that could have influenced its outcome.
